# Genomic epidemiology of SARS-CoV-2 in Cambodia, January 2020 to February 2021

**DOI:** 10.1093/ve/veac121

**Published:** 2022-12-16

**Authors:** Yvonne C F Su, Jordan Z J Ma, Tey Putita Ou, Leakhena Pum, Sidonn Krang, Philomena Raftery, Michael H Kinzer, Jennifer Bohl, Vanra Ieng, Vannda Kab, Sarika Patel, Borann Sar, Wong Foong Ying, Jayanthi Jayakumar, Viseth Srey Horm, Narjis Boukli, Sokhoun Yann, Cecile Troupin, Vireak Heang, Jose A Garcia-Rivera, Yi Sengdoeurn, Seng Heng, Sreyngim Lay, Sophana Chea, Chau Darapheak, Chin Savuth, Asheena Khalakdina, Sowath Ly, Laurence Baril, Jessica E Manning, Etienne Simone-Loriere, Veasna Duong, Philippe Dussart, Ly Sovann, Gavin J D Smith, Erik A Karlsson

**Affiliations:** Programme in Emerging Infectious Diseases, Duke-NUS Medical School, 8 College Rd 169857, Singapore; Programme in Emerging Infectious Diseases, Duke-NUS Medical School, 8 College Rd 169857, Singapore; Virology Unit, World Health Organization COVID-19 Global Referral Laboratory, Institute Pasteur du Cambodge, 5 Preah Monivong Blvd (93), Phnom Penh 12201, Cambodia; Ecole Doctorale GAIA, University of Montpelier, 641 Av. du Doyen Gaston Giraud, Montpellier 34000, France; Virology Unit, World Health Organization COVID-19 Global Referral Laboratory, Institute Pasteur du Cambodge, 5 Preah Monivong Blvd (93), Phnom Penh 12201, Cambodia; Communicable Disease Control Department, Ministry of Health, 80, 289 Samdach Penn Nouth St. (289), Phnom Penh 120407, Cambodia; World Health Organization Country Office, 5 - St 205, Phnom Penh 12355, Cambodia; United States Centers for Disease Control and Prevention, 80, 289 Samdach Penn Nouth St. (289), Phnom Penh 120407, Cambodia; Laboratory of Malaria and Vector Research, US National Institute of Allergy and Infectious Diseases, Phnom Penh, Cambodia; International Center of Excellence in Research, National Institute of Allergy and Infectious Diseases, National Institutes of Health, Phnom Penh, Cambodia; World Health Organization Country Office, 5 - St 205, Phnom Penh 12355, Cambodia; World Health Organization Country Office, 5 - St 205, Phnom Penh 12355, Cambodia; World Health Organization Country Office, 5 - St 205, Phnom Penh 12355, Cambodia; United States Centers for Disease Control and Prevention, 80, 289 Samdach Penn Nouth St. (289), Phnom Penh 120407, Cambodia; Programme in Emerging Infectious Diseases, Duke-NUS Medical School, 8 College Rd 169857, Singapore; Programme in Emerging Infectious Diseases, Duke-NUS Medical School, 8 College Rd 169857, Singapore; Virology Unit, World Health Organization COVID-19 Global Referral Laboratory, Institute Pasteur du Cambodge, 5 Preah Monivong Blvd (93), Phnom Penh 12201, Cambodia; Virology Unit, World Health Organization COVID-19 Global Referral Laboratory, Institute Pasteur du Cambodge, 5 Preah Monivong Blvd (93), Phnom Penh 12201, Cambodia; Virology Unit, World Health Organization COVID-19 Global Referral Laboratory, Institute Pasteur du Cambodge, 5 Preah Monivong Blvd (93), Phnom Penh 12201, Cambodia; Virology Unit, World Health Organization COVID-19 Global Referral Laboratory, Institute Pasteur du Cambodge, 5 Preah Monivong Blvd (93), Phnom Penh 12201, Cambodia; Sequencing Mini-Platform, Institut Pasteur du Cambodge, 5 Preah Monivong Blvd (93), Phnom Penh 12201, Cambodia; Naval Medical Research Unit TWO, Lot#: 80, 289 Samdach Penn Nout, Phnom Penh 120407, Cambodia; Naval Medical Research Unit TWO, Lot#: 80, 289 Samdach Penn Nout, Phnom Penh 120407, Cambodia; Communicable Disease Control Department, Ministry of Health, 80, 289 Samdach Penn Nouth St. (289), Phnom Penh 120407, Cambodia; Communicable Disease Control Department, Ministry of Health, 80, 289 Samdach Penn Nouth St. (289), Phnom Penh 120407, Cambodia; Laboratory of Malaria and Vector Research, US National Institute of Allergy and Infectious Diseases, Phnom Penh, Cambodia; Laboratory of Malaria and Vector Research, US National Institute of Allergy and Infectious Diseases, Phnom Penh, Cambodia; National Institute for Public Health, Lot#: 80, 289 Samdach Penn Nouth St (289), Phnom Penh 120407, Cambodia; National Institute for Public Health, Lot#: 80, 289 Samdach Penn Nouth St (289), Phnom Penh 120407, Cambodia; World Health Organization Country Office, 5 - St 205, Phnom Penh 12355, Cambodia; Epidemiology and Public Health Unit, Institute Pasteur du Cambodge, 5 Preah Monivong Blvd (93), Phnom Penh 12201, Cambodia; Direction, Institute Pasteur du Cambodge, 5 Preah Monivong Blvd (93), Phnom Penh 12201, Cambodia; Laboratory of Malaria and Vector Research, US National Institute of Allergy and Infectious Diseases, Phnom Penh, Cambodia; Department of Virology, Institute Pasteur, 25-28 Rue du Dr Roux, Paris 75015, France; Virology Unit, World Health Organization COVID-19 Global Referral Laboratory, Institute Pasteur du Cambodge, 5 Preah Monivong Blvd (93), Phnom Penh 12201, Cambodia; Virology Unit, World Health Organization COVID-19 Global Referral Laboratory, Institute Pasteur du Cambodge, 5 Preah Monivong Blvd (93), Phnom Penh 12201, Cambodia; Communicable Disease Control Department, Ministry of Health, 80, 289 Samdach Penn Nouth St. (289), Phnom Penh 120407, Cambodia; Programme in Emerging Infectious Diseases, Duke-NUS Medical School, 8 College Rd 169857, Singapore; Centre for Outbreak Preparedness, Duke-NUS Medical School, 8 College Rd, Singapore 169857, Singapore; SingHealth Duke-NUS Global Health Institute, SingHealth Duke-NUS Academic Medical Centre, 8 College Rd 169857, Singapore; Duke Global Health Institute, Duke University, 310 Trent Dr, Durham, NC 27710, USA; Virology Unit, World Health Organization COVID-19 Global Referral Laboratory, Institute Pasteur du Cambodge, 5 Preah Monivong Blvd (93), Phnom Penh 12201, Cambodia

**Keywords:** COVID-19, phylogeny, genetic diversity, coronavirus, pandemic

## Abstract

The first case of coronavirus disease 2019 (COVID-19) in Cambodia was confirmed on 27 January 2020 in a traveller from Wuhan. Cambodia subsequently implemented strict travel restrictions, and although intermittent cases were reported during the first year of the COVID-19 pandemic, no apparent widespread community transmission was detected. Investigating the routes of severe acute respiratory coronavirus 2 (SARS-CoV-2) introduction into the country was critical for evaluating the implementation of public health interventions and assessing the effectiveness of social control measures. Genomic sequencing technologies have enabled rapid detection and monitoring of emerging variants of SARS-CoV-2. Here, we detected 478 confirmed COVID-19 cases in Cambodia between 27 January 2020 and 14 February 2021, 81.3 per cent in imported cases. Among them, fifty-four SARS-CoV-2 genomes were sequenced and analysed along with representative global lineages. Despite the low number of confirmed cases, we found a high diversity of Cambodian viruses that belonged to at least seventeen distinct PANGO lineages. Phylogenetic inference of SARS-CoV-2 revealed that the genetic diversity of Cambodian viruses resulted from multiple independent introductions from diverse regions, predominantly, Eastern Asia, Europe, and Southeast Asia. Most cases were quickly isolated, limiting community spread, although there was an A.23.1 variant cluster in Phnom Penh in November 2020 that resulted in a small-scale local transmission. The overall low incidence of COVID-19 infections suggests that Cambodia’s early containment strategies, including travel restrictions, aggressive testing and strict quarantine measures, were effective in preventing large community outbreaks of COVID-19.

## Introduction

Severe acute respiratory coronavirus 2 (SARS-CoV-2), the causative agent of coronavirus disease 2019 (COVID-19), continues to cause infections globally. On 30 January 2020, the World Health Organization (WHO) first declared the outbreak a Public Health Emergency of International Concern and upgraded it to a pandemic on 11 March 2020 (World Health Organization 2020). As of 4 June 2022, over 531 million laboratory COVID-19 cases have been confirmed with more than 6.3 million deaths reported worldwide (Dong, Du, and Gardner 2020).

SARS-CoV-2 is a positive-sense single-stranded RNA virus of approximately 29,800 nucleotides. The hallmark of SARS-CoV-2 virus is its unprecedented pace of lineage dynamics and expansion among humans, leading to recurrent epidemics (du Plessis et al. 2021). The SARS-CoV-2 virus possesses a distinctive polybasic cleavage site on the spike glycoprotein, which is clearly distinguished from the other four human coronaviruses hCoV-229E, hCoV-OC43, hCoV-NL63, and hCoV-HKU1 (Andersen et al. 2020). Since its emergence in late 2019, the virus evolved into new genetic variants with varying degrees of transmissibility and severity compared to original Wuhan strain of SARS-CoV-2 virus (Kirby 2021; Yadav et al. 2021), resulting in successive epidemic variant-specific waves. Through tracking the evolution and spread of SARS-CoV-2 virus, the scientific community adopted a nomenclature system to systematically designate new lineages for different variants based on nucleotide divergence across the genome, enabling rapid identification of emerging variants (Rambaut et al. 2020). As new virus variants continue to evolve and pose potential public health threats, continuous whole genome sequencing of SARS-CoV-2 is vital to monitor novel constellations of viral genetic mutations and their respective amino acid changes that may influence transmission, virulence, or disease severity.

In Cambodia, public health authorities detected the first confirmed case of SARS-CoV-2 on 27 January 2020 in a traveller from Wuhan, China. In the first 100 days of the pandemic, decisive action in the country’s pandemic preparedness and public health systems helped to contain the threat of escalating COVID-19 outbreaks. Here, we aim to understand the origin and diversity of SARS-CoV-2 viruses detected in Cambodia during the first year of the pandemic, from the first case in January 2020 to mid-February 2021 when widespread community transmission was first detected, and to untangle the epidemiology of small community outbreaks within the capital city, Phnom Penh. Genomic epidemiology of SARS-CoV-2 revealed multiple independent introductions of genetically diverse SARS-CoV-2 lineages into Cambodia through both land borders and air passengers from multiple countries.

## Materials and methods

### Sample collection and viral extraction

Nasopharyngeal and oropharyngeal swabs (combined into one tube) were collected from international passengers upon arrival and locally suspected and contact infections. Extraction of samples was performed with the QIAamp Viral RNA Mini Kit (Qiagen #52906) as described by the manufacturer. Extracted RNA samples were tested via real-time polymerase chain reaction (RT-PCR) for SARS-CoV-2 detection using published methods from Charité Virology (Corman et al. 2020), Hong Kong University, and Insitut Pasteur Paris (Organization WH 2020) as available early in the COVID-19 response. Laboratory report forms completed at the time of sampling contained information on travel history, symptoms, and potential contacts.

### Next-generation sequencing

For early cases, next-generation sequencing libraries were prepared directly from viral RNA extracted from the samples. Complementary DNA was converted to Illumina libraries using the NEBNext Ultra II DNA Library Prep Kit (E7645) according to the manufacturer’s recommendations. Library size and concentration were determined using the 4150 Tapestation system (Agilent, MA, USA). Samples were then sequenced on an Illumina iSeq100 or MiSeq instrument using 150/250 nucleotide paired-end sequencing. Later in 2020, Institut Pasteur du Cambodge established a highly multiplexed PCR amplicon approach (Quick et al. 2017) using the ARTIC Network multiplex PCR primers set v3 (https://artic.network/ncov-2019), with modification as suggested in Itokawa et al. (2020) on Oxford Nanopore GridION/MinION technology. Samples were multiplexed using Oxford Nanopore barcodes and run in batches of 12–24 on a single flow cell. Negative controls were included at each step from extraction to sequence to control for any contamination, and no contamination was detected. Base calling was performed using MinKNOW software. Subsequently, sequences were demultiplexed using Porechop (Wick et al. 2017), quality-trimmed using Nanopolish (Loman, Quick, and Simpson 2015) and BBDuk (https://sourceforge.net/projects/bbmap/), and assembled via MiniMap2 (Li 2018) in Geneious Prime (Biomatters Ltd, New Zealand) against the standard reference strain Wuhan-Hu-1 NC_045512.2. Several viruses were (re-)sequenced for confirmation between matched isolates and clinical samples. All sequences were checked manually for error-prone sites as described at https://virological.org/t/issues-with-sars-cov-2-sequencing-data/473. Lineages and specific mutations were determined from consensus sequences using Nextclade (https://clades.nextstrain.org/) and PANGOLIN (https://pangolin.cog-uk.io/) webservers. Complete genomes of SARS-CoV-2 virus from Cambodia were submitted to GISAID as soon as they were generated (Elbe and Buckland-Merrett 2017).

### Phylogenetic analyses

A total of fifty-four full genomes of SARS-CoV-2 from Cambodia were generated between February 2020 and February 2021. Lineage classifications were assigned to each isolate using Pangolin v3.1.5 (Rambaut et al. 2020) with pangoLEARN v.1.2.36 (O’Toole et al. 2021). To understand the origins and early transmission patterns of Cambodian viruses, phylogenetic analyses were performed in combination with global sequences. We downloaded approximately 972,000 complete SARS-CoV-2 genomes, sampling lineages that comprised the corresponding lineages of Cambodian isolates and global sequences sampled from dominant lineages circulating during that time. These sequences were downloaded in FASTA files from the GISAID database (Shu and McCauley 2017) [retrieved on 14 May 2021]. This dataset consisted of fifty-five Pango lineages, including six A lineages (A.1, A.2, A.23, A.23.1, A.5, and A.6), forty-three B lineages (B, B.1, B.1.1, B.1.1.142, B.1.1.207, B.1.1.214, B.1.1.277, B.1.1.302, B.1.1.317, B.1.1.318, B.1.1.519, B.1.1.7, B.1.160, B.1.177, B.1.177.15, B.1.177.21, B.1.2, B.1.214, B.1.221, B.1.258, B.1.351, B.1.351.2, B.1.351.3, B.1.36.16, B.1.367, B.1.400, B.1.427, B.1.429, B.1.468, B.1.524, B.1.525, B.1.526, B.1.596, B.1.603, B.1.617.1, B.1.617.2, B.1.617.3, B.1.618, B.1.620, B.1.623, B.6, B.6.6, and B.61), one D lineage (D.2), and five P lineages (P.1, P.1.1, P.1.2, P.3, and P.4). Each lineage was randomly sampled to 50–200 taxa to allow the analyses to run within a computationally tractable time. Multiple sequence alignment of SARS-CoV-2 genomes was performed using MAFFT (Katoh et al. 2002) as implemented in Geneious PRIME (Kearse et al. 2012), and any outliers were removed. The final dataset of 3,021 genome sequences was analysed by maximum likelihood (ML) using IQ-TREE 2 (Minh et al. 2019) with the General Time Reversible  + I + G nucleotide substitution model as the best-fit nucleotide substitution model according to the Akaike information criterion in ModelFinder (Kalyaanamoorthy et al. 2017), and statistical support was estimated with 1,000 ultrafast bootstrap replicates (Minh, Nguyen, and von Haeseler 2013). Lineage designations were assigned to each isolate using Pangolin v3.1.5 as described, and the ML tree was visualized and annotated using the *ggtree* package in R (Yu et al. 2017). To estimate the timing of introduction of the A.23.1 variant into Cambodia, we analysed the Cambodian A.23.1 genomes with global A.23 and A.23.1 genomes that were publicly available. Dated phylogenies (*n* = 339) of A.23 and A.23.1 lineages were estimated using a strict clock model in BEAST v1.10.4 (Suchard et al. 2018). Two independent runs of 100 million generations were performed, sampling every 10,000 generations. The convergence of Markov Chain Monte Carlo (MCMC) runs was checked using Tracer v.1.7.1 (Rambaut et al. 2018) after excluding 10 per cent burn-in values to ensure that the effective sampling size values were >200 for all parameters. The MCMC runs were then summarized using LogCombiner, and a maximum clade creditability (MCC) tree was generated in TreeAnnotator.

### Ethics statement

All sequences and information included in this analysis were obtained as part of the first-line testing, analysis, and preparedness of suspected cases through the national outbreak response as part of routine public health and surveillance activities. No personal identifying information or any other individual-specific information was utilized in these studies or manuscript.

## Results

### SARS-CoV-2 cases in Cambodia: January 2020 to February 2021

Following the detection of a cluster of pneumonia cases of unknown aetiology in Wuhan, China, in December 2019, the Cambodian Ministry of Health organized screening of passengers on incoming flights to Cambodia and began testing suspected COVID-19 samples by early January 2020 using standard WHO-provided SARS-CoV-2 protocols. The first COVID-19 case in Cambodia was confirmed on 27 January 2020 in a traveller from Wuhan (Manning et al. 2020). Total sequencing up to 17 February 2021 covered 77 (17.5 per cent) of the 438 cases of COVID-19 detected in Cambodia.

**Figure 1.**
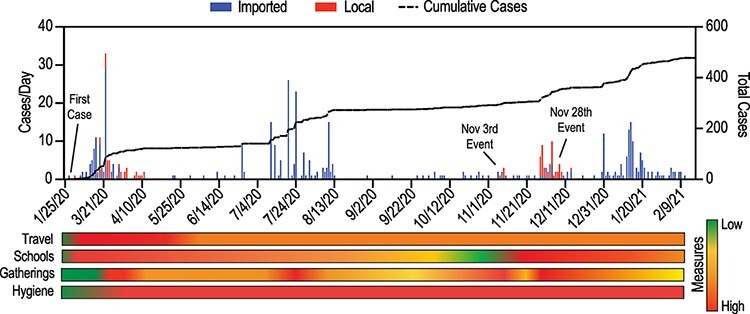
Official RT-PCR confirmed COVID-19 cases in Cambodia between January 2020 and February 2021 and public health response measures. Imported and local cases are shown between January 2020 and February 2021 with cumulative cases (dashed line). Public health measures are indicated by semi-quantitative score based on restrictions based on qualitative assessment and indicated as lowest ( none, 0) to highest (strict, 4) based on implementation and easing dates.

To confirm that COVID-19 was not present before January 2020, all samples collected from the influenza-like illness (ILI)/severe acute respiratory illness (SARI) surveillance system from the end of November 2019 to early January 2020 (*n* = 161) were tested for SARS-CoV-2 with no detections. During 2020 to early 2021, the Cambodian COVID-19 response strategy focused on identifying all cases, including imported and community infections. In addition to mandatory land and air border screening, Cambodian authorities implemented exhaustive contact tracing and management around all confirmed cases. SARS-CoV-2 was actively screened in all individuals meeting the suspect case definition at screening centres, all close contacts of confirmed cases, samples collected through ILI and SARI sentinel surveillance, and patients admitted with pneumonia at hospitals through active case finding. Mass testing was also performed in certain settings (e.g. apartment buildings, banks, and shops) to assess community spread if community cases were detected. As of 15 February 2021, 238,963 individuals were tested using RT-PCR from a total of 361,607 tests, with a positivity rate of 0.2 per cent. In addition, 4,719 ILI/SARI sentinel surveillance specimens tested negative for SARS-CoV-2 in this time period (WPRO-Cambodia 2021). Therefore, limited local SARS-CoV-2 transmission was observed in Cambodia during early and late 2020, with no local transmission detected between April and October 2020, partially due to the country’s rapid mitigation strategies including stricter travel restrictions and quarantine measures (Nit et al. 2021) (Fig. 1).

### Early 2020: tourists and pilgrims (introductions of multiple SARS-CoV-2 lineages)

**Figure 2.**
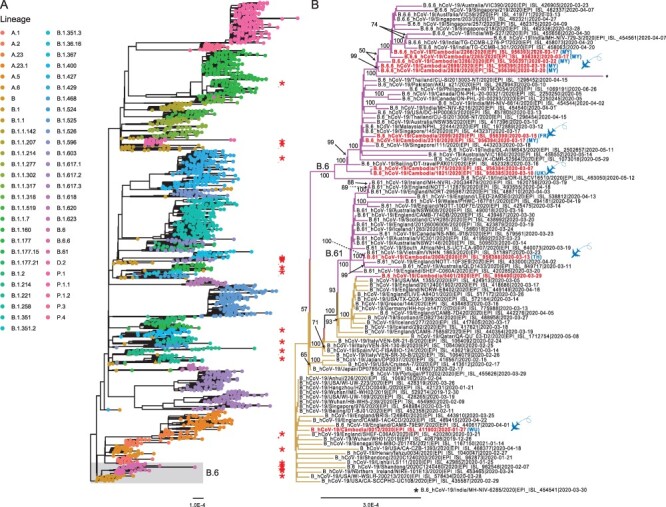
SARS-CoV-2 genetic diversity in Cambodia during January 2020–February 2021 in the global context. (A) ML phylogeny of SARS-CoV-2 genomes inferred using IQ-TREE 2. The tree was rooted with the Wuhan/IPBCAMS-WH-01/2019 virus. Coloured tip circles represent different lineages as inferred by pangolin software. Shaded area indicates the B.6 lineage; asterisks represent SARS-CoV-2 genomes from Cambodia. The scale bar indicates the number of substitutions per site. (B) Evolutionary relationship of B.6 lineage (highlighted in grey box in A). Purple branches denote B6/B.61 lineage viruses. Highlights denote past travel history of infected individuals from other geographical regions into Cambodia. Abbreviations: FR, France; MY, Malaysia; TH, Thailand; Wu, Wuhan. The flying airplane symbol represents air travel. Bootstrap values greater than 50 per cent are indicated at the major nodes in the fully resolved tree.

Following the first COVID-19 case in Cambodia in late January 2020, sporadic transmissions of SARS-CoV-2 virus were detected, but they were quickly contained by rapid mitigation controls, including strict quarantine, school closure, and mandatory testing of all incoming travellers and suspected cases. Starting in March 2020, COVID-19 cases were primarily detected in international airline passengers entering Cambodia. Phylogenetic analyses of globally sampled genomes indicated that the first Cambodian isolate (hCoV-19/Cambodia/0012/2020) was situated in the basal part of the tree (Fig. 2A). This sequence shared the highest nucleotide similarity (99.98 per cent) to an early Wuhan COVID-19 patient (hCoV-19/Wuhan/IPBCAMS-WH-01/2019) sampled on 24 December 2019 (Fig. 2B).

The inferred evolutionary tree indicates that subsequent viruses from Cambodia (marked by asterisks in Fig. 2A) were interspersed throughout different phylogenetic lineages of SARS-CoV-2. Between February and April 2020, the majority of SARS-CoV-2 viruses detected in Cambodia grouped with lineage B viruses. In this early B lineage (Fig. 2B), several individuals attended a religious meeting in Malaysia and were gathered together on 28 February 2020. They arrived in Cambodia on 11 March 2020 and tested positive for SARS-CoV-2 approximately 1 week after arrival. Two cases sequenced from this cluster (hCoV-19/Cambodia/2265/2020 and hCoV-19/Cambodia/2266/2020) were closely related and grouped as B.6.6 lineage. They had direct contact with two local individuals whom had no travel history who were also infected. As expected, the viruses from the two local cases (hCoV-19/Cambodia/2928/2020 and hCoV-19/Cambodia/3286/2020) clustered together with the two travellers from Malaysia, indicative of transmission or exposure to the same source.

**Figure 3.**
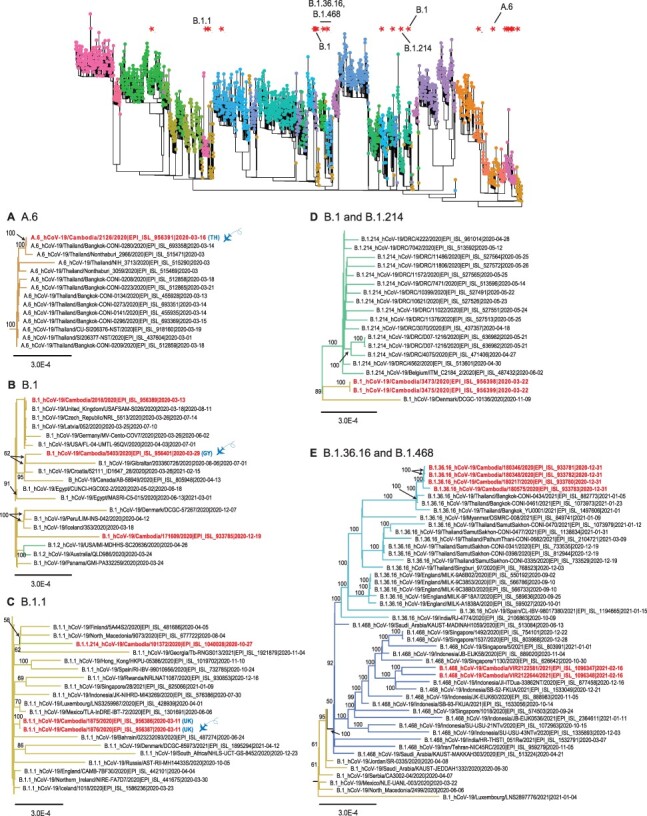
Evolutionary relationships of SARS-CoV-2 lineages in Cambodia from March 2020 to February 2021. The top panel displays the global ML tree as shown in Fig. 1. The bottom panel shows the sub-trees to indicate inferred phylogenetic positions of Cambodia viruses in (A) A.6 lineage, (B) B.1 lineage, (C) B.1.1 lineage, (D) B.1.214 lineage, and (E) B.1.36.16 and B.1.468 lineages. Highlights denote past travel history of infected individuals from other geographical regions into Cambodia. Abbreviations: GY, Guyana; TH, Thailand;. The flying airplane symbol represents air travel. Bootstrap values greater than 50 per cent are indicated at the major nodes.

Early in 2020, multiple independent introductions of SARS-CoV-2 virus into Cambodia were observed, mainly due to the entry of individual travellers through international flights. They arrived in Cambodia from different countries such as France (hCoV-19/Cambodia/2099/2020), Malaysia (hCoV-19/Cambodia/2690/2020), and Thailand (hCoV-19/Cambodia/2004/2020 and hCoV-19/Cambodia/2126/2020) and tested SARS-CoV-2-positive (Figs 2B, 3A). Phylogenetic inference indicated that these sequences were scattered through the tree, and they belonged to both A (e.g. A.6) and B lineages (e.g. B.6 and B.61). These lineages were widely spread across diverse geographical regions, including Europe (e.g. England, Iceland, and Wales), South East Asia (e.g. Malaysia, Singapore, and Thailand), South Asia (e.g. India and Pakistan), North America (Canada and USA), and Oceania (e.g. Australia).

Starting from March 2020, several B.1 and B.1.1 viruses were also identified in Cambodia following independent introductions (Fig. 3B–D). In particular, three passengers from a group travelled from Europe on 2 March 2020 for a cruise in Vietnam before their arrival in Cambodia on 6 March 2020. Although all three were confirmed with SARS-CoV-2 and shared the same travel history, we identified one sample (hCoV-19/Cambodia/1821/2020) that belongs to B.6 lineage (Fig. 2B), whereas the other two (hCoV-19/Cambodia/1875/2020 and hCoV-19/Cambodia/1876/2020) clustered together within the B.1.1 lineage (Fig. 3C), indicating infection from different sources despite a shared travel history.

### Late 2020: border vigilance and disentangling clusters

**Figure 4.**
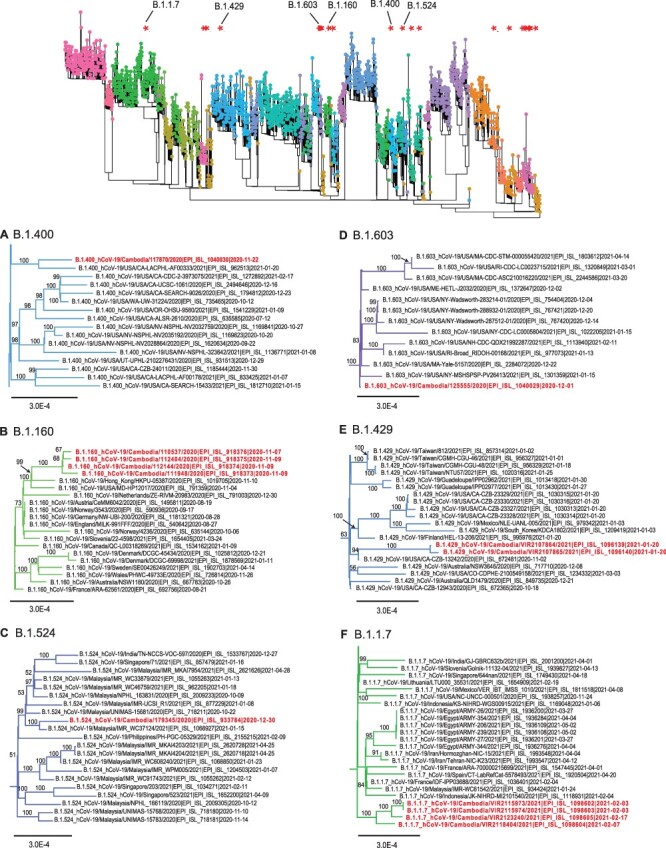
Evolutionary relationships of different SARS-CoV-2 variants of concern or significance in Cambodia. The top panel displays the global ML tree as shown in Fig. 1. The bottom panel shows the sub-trees to indicate inferred phylogenetic positions of Cambodia viruses in (A) B.1.400 lineage, (B) B.1.160 lineage, (C) B.1.524 lineage, (D) B.1.603 lineage, (E) B.1.429 lineage, and (F) B.1.1.7 lineage. Bootstrap values greater than 50 per cent are indicated at the major nodes.

**Figure 5.**
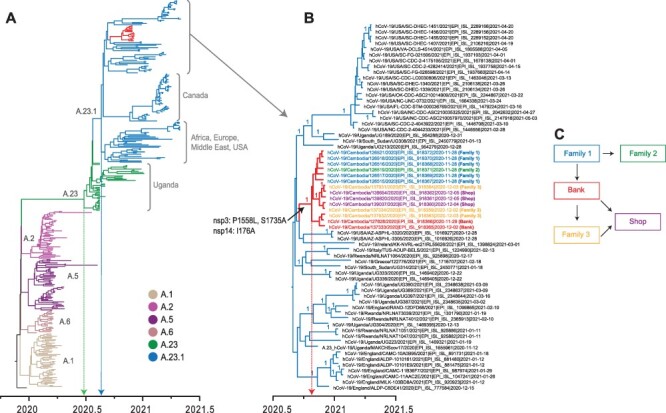
Temporal phylogeny of A.23 variants and related SARS-CoV-2 lineage A sequences (*n* = 339). (A) The MCC tree was reconstructed based on the whole genome of SARS-CoV-2 from Cambodia and globally. Coloured branches represent different lineage A viruses. Line indicates the mean TMRCA estimate of A.23 lineage. (B) The estimated mean TMRCA of Cambodia A.23.1 viruses is indicated. Three non-synonymous amino acid mutations unique to A.23.1 viruses from Cambodia are noted on the corresponding tree branch. (C) Relationships of A.23.1 virus infection clusters in Cambodia determined through epidemiological investigation.

Following initial introductions in early 2020, very limited local SARS-CoV-2 transmission was observed in Cambodia during April–October 2020, mostly likely due to the country’s rapid implementation of mitigation strategies including travel restrictions and quarantine measures. However, between November 2020 and February 2021, multiple different co-circulating variants of SARS-CoV-2 were introduced into the country. These included B.1.36.16 (Fig. 3E), B.1.468 (Fig. 3E), B.1.400 (Fig. 4A), B.1.160 (Fig. 4B), B.1.524 (Fig. 4C), B.1.603 (Fig. 4D), B.1.429 (Fig. 4E), and B.1.1.7 variants (Fig. 4) as well as A.23.1 variants (Fig. 5). More specifically, viruses of B.1.36.16, B.1.468, and B.1.524 lineages are likely to been introduced from Asian countries including Thailand (likely via land border), Indonesia, and Malaysia (Figs 3E and 5C), whereas B.1.400, B.1.429, and B.1.603 viruses may have been introduced from the USA (Fig. 4). These variants included the B.1.1.7 (referred to as Alpha, or GV/20E.EU1) Variant of Concern (VoC), initially detected in the UK, and the B.1.429 Variant of Interest (referred to as Epsilon, or GH/452R.V1), originally detected in the USA. Furthermore, limited local clusters were detected in Cambodia (Figs 3E, 4B, 4F, and 5), indicating short chains transmission of SARS-CoV-2 within the country.

### November 3^rd^ Event: cluster associated with quarantine waiver (B.1.160)

In early November 2020, a foreign dignitary visited Cambodia; however, given the nature of their visit, the standard 14-day quarantine requirement was waived. Upon leaving Cambodia, the dignitary arrived in Thailand and was documented by RT-PCR as SARS-CoV-2-positive (Mungaomklang et al. 2021). Subsequent testing of close contacts in Cambodia revealed several more cases in this cluster, consisting of a group of viral sequences sharing a high level of nucleotide identity (99.99 per cent), indicating a single source of introduction into Cambodia (Fig. 4B).

### November 28^th^ event: phylogenetic cluster of A.23.1 virus in Cambodia

On 28 November 2020, an individual with respiratory symptoms but with no history of international travel tested positive for COVID-19. Extensive contact tracing of contacts resulted in testing 41,000 samples from more than 19,000 individuals. Over the next 2 weeks, a total of forty-one cases directly or secondarily linked to the index case were detected, making it Cambodia’s largest community cluster to date in 2020. These cases were linked to the putative index case and composed of the index’s family members, the family of the driver working for individuals in the household of the index case, individuals working in a bank, and, subsequently, several retail outlets. Genomic sequencing of these positive cases identified viruses as A.23.1 variant (Fig. 5A), directly unlinking it from the November 3^rd^ event (above).

The A.23.1 sublineage diverged soon after the emergence of A.23 lineage in Uganda in August 2020. Since October 2020, the A.23.1 sublineage spread to Canada, Europe, Middle East, and the USA. We retrieved fourteen complete genomes of A.23.1 viruses from Cambodia and analysed them with all A.23.1 global sequences that are publicly available. Our results indicated that all fourteen Cambodian A.23.1 sequences were distinctly grouped (denoted by branches in Fig. 5A). These viruses were well-nested within the A.23.1 sublineage (denoted in Fig. 5A). Here, we inferred the timing of A.23.1 variant introduction into Cambodia. Our temporal phylogeny (Fig. 5) indicates that the mean time to most common ancestor (TMRCA) of the entire A.23.1 sublineage (dotted blue line in Fig. 5A) was estimated around 1 August 2020. Specifically, the mean TMRCA of Cambodian A.23.1 clade (dotted red line in Fig. 5B) was estimated around 24 October 2020. These Cambodian A.23.1 sequences were closely related to viruses from Africa, Europe, and USA. The ancestral A.23 virus may have emerged around 25 June 2020 (dotted green line in Fig. 5B), approximately 2 months before its first detection in Uganda. All fourteen Cambodia A.23.1 viruses clustered together in a strongly supported monophyletic clade (posterior probability  = 1.00), indicating a single introduction followed by community transmission. Phylogenetic analysis recovered the link between the two family clusters at the centre of this outbreak, Cluster A (Family 1) and B (Family 2), which formed an unsupported monophyletic group (Fig. 5C). The viruses from Cluster C (Bank) and Cluster D (Family 3) also formed a group, while Cluster D and E (Retail) viruses were similarly grouped. While contact tracing contributed to elucidation of this cluster, the utility of genetic analysis in linking clusters was essential for characterization and containment of this outbreak.

### Early 2021: watching for alpha VoC

Cambodia remained vigilant to the introduction of Alpha VoC (PANGO lineage B.1.1.7), first detected in the UK in September 2020, and increased genomic surveillance to prevent its entry (Davies et al. 2021a, 2021b). The first case of Alpha infection detected in a traveller was confirmed on 3 February 2021 and other local Alpha variant cases over the next 2 weeks shared 99.99–100 per cent nucleotide identities. Phylogenetic analysis of Alpha variants detected in Cambodia showed no clear association with viruses from any particular geographic region (Fig. 4F).

## Discussion

In this study, we describe the introduction of SARS-CoV-2 virus into Cambodia during the first year of the COVID-19 pandemic. The first detected case was a traveller from Wuhan, China, with a complete virus genome closely resembling the prototype Wuhan SARS-CoV-2 genome. At the beginning of a pandemic, a fundamental concern is the ability of lower-income countries to respond to the immense public health and socioeconomic challenges due to the limited supply of resources and infrastructure. The investments in preparedness and rapid development of response plans in countries such as Cambodia were crucial in battling COVID-19. Immediately following the first detection of SARS-CoV-2, rapid response from the government and regional partners allowed quick adoption of detection protocols and rigorous testing of all suspected cases. During the pandemic, Cambodia further enhanced technical capabilities for early identification and isolation of confirmed cases and improved sequencing capacities to allow the prompt release and sharing of viral sequences.

From the very beginning of the pandemic, Cambodia imposed strict screening procedures at borders and comprehensive PCR testing was conducted for all travellers upon arrival and for close contacts of COVID-19 cases. To accurately identify and track the viruses entering Cambodia, genome sequencing was performed from positive SARS-CoV-2 samples as available throughout the study period. We demonstrate that the first year of SARS-CoV-2 cases in Cambodia was dominated by multiple importations via international air travel from various regions such as Asia, Africa, and Europe, with at least seventeen different PANGO lineages identified. Our phylogenetic analyses revealed that most of the SARS-CoV-2 genomes from Cambodia closely resembled sequences from viruses detected from the cities or countries where the travellers boarded flights, resulting in a genetically diverse constellation of virus from PANGO A and B lineages. While the two lineages continue to evolve and differentiate into successive sub-lineages, Cambodia quickly detected and identified these new variants at the airport and land borders soon after their emergence. However, with a limited number of cases, it is difficult to determine the exact source of each detection.

During the first year of the COVID-19 pandemic, the spread of SARS-CoV-2 was successfully contained via strict enforcement of quarantine at designated facilities. This resulted in limited to minimal household or workplaces outbreaks, and there were no large transmission events detected in any major province. Notably, a limited A.23.1 outbreak occurred in November 2020, with at least fourteen cases reported in Phnom Penh. The A.23.1 viruses were derived from A.23 lineage viruses that initially emerged in two prison outbreaks in Uganda in August 2020 (Bugembe et al. 2021). By October 2020, the A.23 variant had evolved into the A.23.1 lineage that became dominant throughout Uganda, accounting for 90 per cent of all SARS-CoV-2 viruses detected in the country. A.23.1 viruses then rapidly spread into the rest of Africa and then in Europe, Canada, the Middle East, and the USA. The A.23.1 virus outbreak in Phnom Penh was successfully contained by December 2020. The lineage has not been detected in global surveillance since early July 2021, indicating that it is no longer circulating in human populations. Towards the end of our study period, we also detected the Alpha VoC (B.1.1.7) that was initially detected in the UK in September 2020 and had increased (Davies et al. 2021a). The emergence of the Alpha VoC led to a significant spike in global infections and mortality (Lyngse et al. 2021; Volz et al. 2021; Davies et al. 2021a, 2021b), eventually becoming the dominant SARS-CoV-2 variant in over 114 countries by early 2021 (Lyngse et al. 2021; Volz et al. 2021; Davies et al. 2021b).

Through the first year of pandemic, Cambodia detected only 478 confirmed cases of SARS-CoV-2 infection, indicating that Cambodia’s mitigation plans and public health capacities contributed to the successful control of the pandemic. These mitigating public health and social measures included rapid surveillance including contact tracing, aggressive testing, stringent land border arrangements, air travel restrictions, strict quarantine protocols, social group size limits, and successful movement restrictions in addition to basic individual measures such as mask wearing, physical distancing, ventilation, and hand and respiratory hygiene following WHO guidelines. Neighbouring countries, such as Laos, Thailand, and Vietnam, also had low incidences of COVID-19 cases due to the quick implementation of countermeasures and efficient screening and isolation of confirmed cases, similar to measures taken in Cambodia (Issac et al. 2021; Manabe et al. 2021; Phonvisay et al. 2021; Virachith et al. 2021). Therefore, despite concerns of lower-income countries being able to respond to a pandemic, Cambodia had proven successful in preventing the importation and localized spread of SARS-CoV-2 during the early phase of COVID-19 pandemic. Furthermore, effective disease surveillance and response, and the rapid sharing of genomic data, served a critical role in understanding the evolution and transmission of SARS-CoV-2 virus in Cambodia and in informing government mitigation and control measures.

## Supplementary Material

veac121_Supp

## Data Availability

The data from this study were deposited in GISAID as it were generated to facilitate the pandemic response globally. All data generated or analysed during this study are included in this published article (and its Supplementary Information files).
